# Determination of *Listeria monocytogenes* Growth during Mushroom Production and Distribution

**DOI:** 10.3390/foods2040544

**Published:** 2013-11-27

**Authors:** Dara Leong, Avelino Alvarez-Ordóñez, Floriane Guillas, Kieran Jordan

**Affiliations:** Teagasc Food Research Centre, Moorepark, Fermoy, Co. Cork, Ireland; E-Mails: dara.leong@teagasc.ie (D.L.); avelino.alvarez-ordonez@teagasc.ie (A.A.-O.); floriane.guillas@sfr.fr (F.G.)

**Keywords:** *Listeria monocytogenes*, mushrooms, growth, challenge tests, food safety

## Abstract

In the EU, food is considered safe with regard to *Listeria monocytogenes* if its numbers do not exceed 100 CFU/g throughout the shelf-life of the food. Therefore, it is important to determine if a food supports growth of *L. monocytogenes*. Challenge studies to determine the ability of a food to support growth of *L. monocytogenes* are essential as predictive modelling often overestimates the growth ability of *L. monocytogenes*. The aim of this study was to determine if growth of *L. monocytogenes* was supported during the production and distribution of mushrooms. A three-strain mixture of *L. monocytogenes* was inoculated onto three independent batches of whole mushrooms, sliced mushrooms, mushroom casing and mushroom substrate at a concentration of about 100–1000 CFU/g. The batches were incubated at potential abuse temperatures, as a worst case scenario, and at intervals during storage *L. monocytogenes* numbers, % moisture and pH were determined. The results showed that the sliced and whole mushrooms had the ability to support growth, while mushroom casing allowed survival but did not support growth. Mushroom substrate showed a rich background microflora that grew on *Listeria* selective media and this hindered enumeration of *L. monocytogenes*. In the case of this study, Combase predictions were not always accurate, indicating that challenge studies may be a necessary part of growth determination of *L.* *monocytogenes*.

## 1. Introduction

Although the incidence of *Listeria monocytogenes* infection is relatively low, listeriosis has the third highest mortality rate of all foodborne pathogens. It is especially dangerous to the immunocompromised; including the young, the elderly, pregnant women and those with pre-existing health issues [1,2]. *L. monocytogenes* is of particular concern in ready-to-eat foods as the heat step of cooking, which would normally kill *L. monocytogenes,* is absent. Listeriosis outbreaks have commonly been linked to dairy, egg, meat and fish products and produce in the past [3,4]. According to the last EU summary report on zoonoses, zoonotic agents and food-borne outbreaks 1476 confirmed human cases of listeriosis were reported in 2011 (0.32 cases per 100,000 population), while listeriosis represented the most severe food borne human disease in terms of hospitalisation and fatal cases (12.7%) [5].

*L.* *monocytogenes* is widespread in the environment so it is not surprising that it is occasionally found in food. The occurrence of <100 CFU/g is permitted, as long as it can be shown that the numbers will not increase during the shelf-life of the food [6]. Predictive modelling based on experiments performed with laboratory media can be used to give an estimate of the ability of a food to support growth of *L. monocytogenes*, but in many cases, growth is predicted where there is actually no growth [7,8,9]. Therefore, challenge studies to determine the ability of a food to support growth of *L. monocytogenes* are essential.

*Agaricus bisporus*, widely available as commercial mushrooms, are grown commercially in a substrate which is prepared in two or three phases. In phase one, the raw materials which make up the substrate (which may contain wheaten straw, horse manure, poultry manure, and gypsum) are mixed together and composted. The composted phase one substrate is then moved to undergo a further heating step (phase two) at a temperature of 58–59 °C for 8–9 h. Following phase two, *A. bisporus* spawn is added to the substrate. In a phase 3 facility the mycelium grows through the substrate for several days (usually <19 days) after which a nutrient supplement may be added prior to its dispatch to the mushroom producer. On a mushroom production unit, a 5 cm layer of casing material (a mixture of peat with crushed limestone or spent sugar beet lime and water) is added on top of the substrate. *A. bisporus* mycelium then grows through the casing for several days before mushrooms appear on the surface of the casing and are then harvested [10]. Mushrooms are usually hand-picked and packaged for sale either whole or sliced.

Although there have been no reports of listeriosis directly attributed to consumption of mushrooms, various recent surveys have demonstrated that *L. monocytogenes* contamination of mushrooms [11] and mushroom production facilities [10] can occur. In 2012, a recall was also issued by The Canadian Food Inspection Agency (CFIA) on sliced white mushrooms potentially contaminated with *L. monocytogenes* [12]. However, there is little information available on whether *L. monocytogenes* is capable of growing during mushroom production and distribution. González-Fandos and co-authors [13] have previously evaluated the potential of *L. monocytogenes* to grow in whole mushrooms packed in two sorts of polymeric films and stored at 4 °C and 10 °C, and they reported growth of between 1 and 2 log units during the first 48 h of incubation.

The objective of the current study was to investigate growth of *L. monocytogenes* on both sliced and whole mushrooms (*A. bisporus*) as well as in mushroom casing and mushroom substrate. 

## 2. Materials and Methods

### 2.1. Sample Collection

Whole and sliced mushrooms, mushroom substrate and mushroom casing (three independent batches of each) were obtained from a mushroom supplier in Ireland. All mushroom samples were transported to the laboratory in chilled containers and immediately placed in a cold room at 4 °C and inoculation was performed within 16 h. Casing and substrate samples were transported to the laboratory and stored at 20 °C for 2 h prior to inoculation.

### 2.2. Assessment of *L. monocytogenes* Natural Contamination

Before inoculation, a sample from each batch was removed and tested by enrichment and enumeration for natural contamination with *L. monocytogenes* using the ISO 11290-1 and the ISO11290-2 methods, except that only Agar *Listeria* acc. to Ottavani & Agosti (ALOA) agar (Biomérieux, UK) was used. If any positive indication of *L. monocytogenes*, *i.e.*, round, green colonies with a halo was detected, analysis of that batch was terminated.

### 2.3. Bacterial Strains, Culture Conditions and Sample Inoculation

A cocktail of three *L. monocytogenes* strains obtained from the *Listeria* Strain collection at Teagasc Food Research Centre, Moorepark was used for each challenge test. The cocktail comprised a clinical isolate, obtained from University College Hospital Galway (number 757), a persistent strain, isolated from a cheese processing plant (number 6179), and a strain previously isolated from an environmental swab from a mushroom production facility (number 958). Cultures of each strain were grown separately overnight in 10 mL Brain Heart Infusion (BHI) broth at 37 °C and mixed together to achieve equal numbers of each strain in the mix used for inoculation. Although the European guidelines indicate that overnight cultures should be incubated at similar temperatures to the test conditions [6], studies have shown that incubation of overnight cultures at optimum temperature gave similar results [14]. For each batch, a separate inoculum cocktail was prepared from independent overnight cultures.

For whole and sliced mushrooms an inoculum of ~10^3^ CFU/mL was used to give an approximate contamination of 10^2^ CFU/g. Three independent batches of mushrooms were inoculated by pouring 500 mL of the inoculum into 200 g of produce, shaking to coat the mushroom surface and leaving to stand for 15 min before pouring off the excess inoculum. Immediately following inoculation, 4 samples were taken from different areas of the batch and cells were enumerated, as described below, to ensure inoculation was even throughout the sample. Innoculated mushroom samples were placed in a plastic tray covered by a polymeric film during storage (to simulate the commercial situation).

Mushroom casing batches were inoculated using a blender. Ten milliliters of a ~10^3^ CFU/mL inoculum was added (by a fine mist spray, with appropriate precautions) to 200 g of mushroom casing and blended for 5 min. Mushroom substrate batches were inoculated similarly by adding 10 mL of a ~10³ CFU/mL inoculum to 200 g of mushroom substrate and mixing by shaking.

### 2.4. Challenge Test Conditions and Cell Enumeration

Inoculated samples were stored at the normal production temperature (mushroom substrate and mushroom casing at 20 °C) or potentially abusive storage temperatures (whole and sliced mushrooms at 8 °C and 15 °C), and samples were taken at predetermined time intervals for cell enumeration, pH and moisture determination. The length of incubation depended on the shelf-life of the product. Both sliced and whole mushrooms were incubated for 10 days, whereas casing and substrate were incubated for 20 days.

Cells were enumerated at regular time points to facilitate predictive modelling. At each sampling point, samples were removed from each batch (5 g for whole and sliced mushrooms and 10 g for casing and substrate), mixed with Maximum Recovery Diluent (MRD) in a 1:5 dilution and blended in a stomacher for 4 min. Following this, 0.5 mL aliquots were spread in duplicate onto ALOA plates. As required, further serial dilutions were performed in MRD and plated similarly onto ALOA. Plates were incubated at 37 °C for 48 h before cells were counted and cell numbers per gram were calculated. Experimental data were fitted using the DMfit tool of ComBase [15]. Combase predictive models were also used to predict bacterial growth under similar conditions to the experimental data [15]. For the predictions of *L. monocytogenes* growth in mushrooms, a temperature of 15 °C, pH of 7 and water activity (a_w_) of 0.99 were used. For the predictions of *L. monocytogenes* growth in mushrooms casing, a temperature of 20 °C, a pH of 7.3 and an a_w_ of 0.985 were used. 

At each sampling point, the pH and moisture content were also determined. To measure the pH of casing and substrate, a 10 g sample from each batch was mixed with 6 mL water and homogenised and a food pH probe was used to measure the pH. The pH of mushrooms was measured similarly except a 20 g sample was homogenised with 12 mL water. To measure moisture content, an aluminium cup was dried for 1 h at 102 °C, then placed in a dessicator for 1 h and weighed. A sample of approximately 1 g from each batch was weighed, correct to 3 decimal places, in the aluminium cup. The sample was dried in a 102 °C oven for 5 h and then placed in a dessicator for 1 h before being weighed again. The weight loss expressed as a percentage of the original weight was calculated and represents the moisture content of the sample.

## 3. Results

### 3.1. Challenge Testing in Mushroom Substrate and Mushroom Casing

Mushroom substrate was found to have a rich background non-*Listeria* microflora which possessed the ability to grow on *Listeria* chromogenic agar plates. This made it impossible to accurately enumerate *L. monocytogenes* colonies on ALOA agar plates. Further experiments with mushroom substrate were not undertaken.

**Figure 1:**
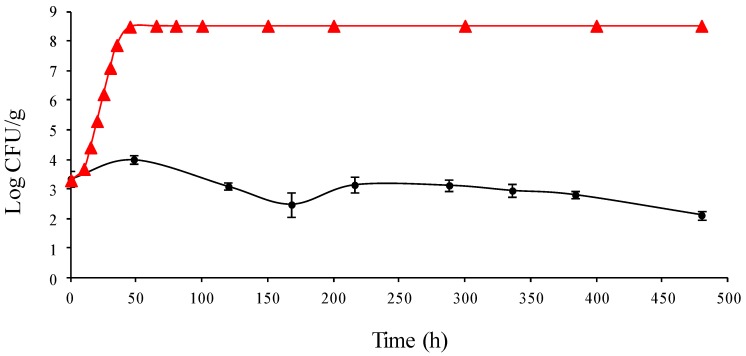
Logarithmic growth of *Listeria monocytogenes* in mushroom casing. The data points used for the observed growth of *L. monocytogenes* are mean values of duplicate analysis of triplicate batches. Growth of *L. monocytogenes* observed (●), growth of *L. monocytogenes* predicted by ComBase (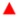).

Regarding mushroom casing challenge tests, *L. monocytogenes* numbers increased by approximately 1 log unit over the first 50 h of incubation at 20 °C (Figure 1). Following this, *L. monocytogenes* numbers progressively decreased to below the original inoculation level. Indeed, at the end of the 20 day incubation period, *L. monocytogenes* was present at ~10^2^ CFU/g (approximately 1 log unit lower than at time zero; Figure 1).

**Figure 2:**
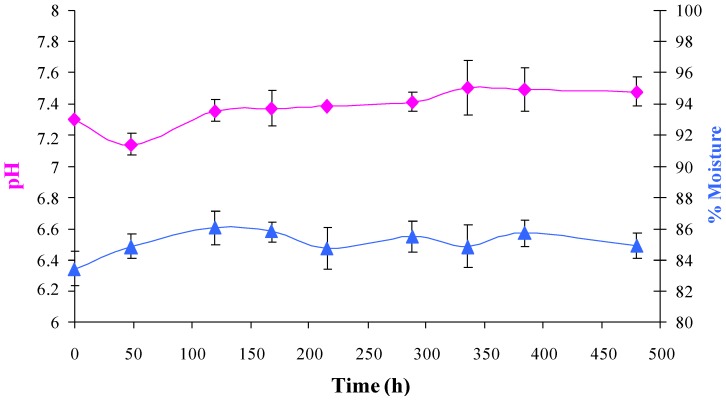
Evolution of pH and % moisture in mushroom casing. The data used are mean values of duplicate analysis of triplicate batches. pH (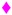), % moisture (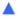).

The moisture and pH of mushroom casing remained almost constant over the course of the experiment (Figure 2). The initial moisture content was 83% and increased slightly to 84%–86%. The pH remained at approximately 7.3 throughout the experiment. 

### 3.2. Challenge Testing with Sliced and Whole Mushrooms

**Figure 3:**
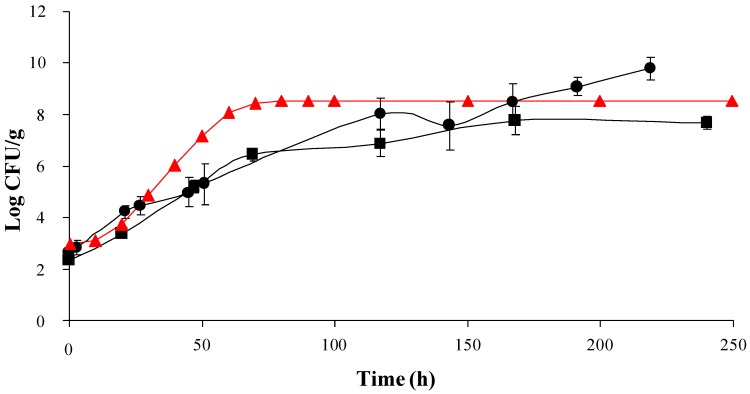
Logarithmic growth of *Listeria monocytogenes* in sliced and whole mushrooms. The data points used for growth of *L. monocytogenes* are mean values of the duplicate analysis of triplicate batches. Growth of *L. monocytogenes* observed in sliced mushrooms (●) growth of *L. monocytogenes* observed in whole mushrooms (■), growth of *L. monocytogenes* predicted by ComBase (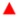).

Growth of *L. monocytogenes* was seen at 15 °C in mushrooms with no substantial difference between sliced and whole mushrooms throughout the majority of the experiment (Figure 3). Fitting of the experimental data to the Baranyi and Roberts model [16] was performed using the DMfit tool of ComBase. No lag phase was observed in either whole mushrooms or sliced mushrooms, while maximum growth rates were 0.04 and 0.06 log CFU/g/h, respectively. Final population densities were 7.3 and 9.5 log CFU/g, respectively. Challenge testing was also carried out in whole mushrooms at 8 °C, and similar increases in bacterial numbers were observed (e.g., in the first 24 h of incubation, the numbers of *L. monocytogenes* increased by more than 0.5 log_10_, which is assumed as the boundary to define whether a food is capable of supporting the growth of *L. monocytogenes*, and final population densities reached 7.9 log CFU/g (data not shown).

**Figure 4:**
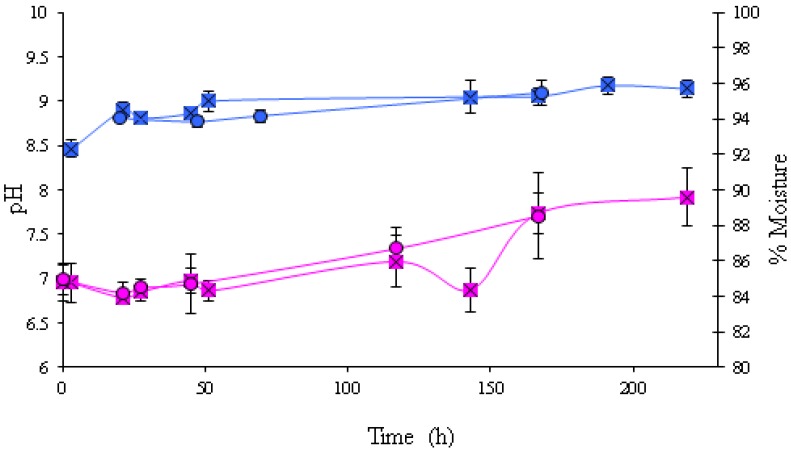
Evolution of pH and % moisture in sliced and whole mushrooms. The data used are mean values of duplicate analysis of triplicate batches. pH of sliced mushrooms (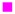), pH of whole mushrooms (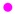), % moisture of sliced mushrooms (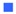), % moisture of whole mushrooms (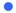).

No remarkable differences were seen in pH or moisture content between sliced and whole mushrooms (Figure 4). The pH increased from approximately 7.0 at the beginning of the experiment to approximately 7.8 at the end of the experiment in both sliced and whole mushrooms. This increase occurred after approximately 150 h of incubation. The moisture content of mushrooms increased from 92% at the initiation of the experiment to 95% at the end of the experiment (Figure 4).

### 3.3. Accuracy of ComBase as a Predictive Tool

The accuracy of the ComBase predictor on-line tool to predict the growth of *L. monocytogenes* in mushrooms and mushroom casing was determined using the pH and temperature conditions prevailing in the experimental challenge studies above described (Figure 1 and Figure 3) as input data. Although a_w_ was not measured in these experiments, the a_w_ used for predictions was chosen based on available literature and a worst case scenario situation considering the variability which can exist in the a_w_ of mushrooms and casing. The ComBase predictor tool anticipated growth of *L. monocytogenes* in both mushrooms and mushroom casing with predicted growth rates of 0.11 log CFU/g/h and 0.14 log CFU/g/h, respectively.

## 4. Discussion

A major determinant factor in the risk of listeriosis is the ability of foodstuffs to support growth of *L. monocytogenes* at normal or reasonable abuse storage temperatures. However, data on the ability of foods to support growth are scarce. In the current study, the growth ability of *L. monocytogenes* was assessed in mushroom casing and whole and sliced mushrooms in an attempt to determine the current risk of *L. monocytogenes* growth throughout mushroom production and distribution. Even though water activity was not measured, the percentage moisture in the matrices demonstrates sufficient water to support growth. Unfortunately, due to the presence within the substrate of a rich background microflora which possessed the ability to grow on *Listeria* chromogenic agar, growth potential assessment in mushroom substrate was not possible using the methodology described. Other alternative methodologies, such as use of genetically modified strains marked with an antibiotic resistance gene cassette, or engineered to emit light, might be followed to overcome this issue.

During mushroom casing incubation, *L. monocytogenes* numbers decreased over the time period tested. However, there was some indication of a small degree of growth at the first days of incubation (Figure 1). This may need to be investigated further. Since the pH (7.3) and water content (83%) conditions prevailing in mushroom casing are not predicted to be lethal for *L. monocytogenes*, the decline in bacterial numbers observed throughout the challenge test could be due to the presence of competitive microflora within mushroom casing which could be responsible for a progressive inactivation of *L. monocytogenes*. It could also be due to other inhibitory factors not measured during the present study including the presence of natural antimicrobial compounds [17] or bacteriocins [18]. Nonetheless, *L. monocytogenes* remained viable at the end of the 20 day period and therefore, if present, could transfer to growing mushrooms.

The increase in *L. moncytogenes* numbers in both whole and sliced mushrooms without an observable lag phase seen in this study (Figure 3) is in agreement with a previous study by González-Fandos *et al.* (2001) [13], who found a 1 and 2 log increase in *L. monocytogenes* numbers in mushrooms within 48 h at 4 °C and 10 °C incubation, respectively. However, these authors observed that, after 48 h, the bacterial population remained relatively stable during incubation from 3 to 8 days, and after day 8 of incubation they reported a decline in bacterial numbers of around 1–2 log units. They linked these findings to the growth characteristics of the competitive microflora present in mushrooms. On the contrary, the results of the current study showed a fast and progressive increase in bacterial numbers until final population densities of 7.3 and 9.5 log CFU/g were reached in whole mushrooms and sliced mushrooms, respectively. Hoelzer and co-authors [19] have recently reviewed the available data on *L. monocytogenes* growth dynamics in produce, and it is important to note that, in terms of growth rate and maximum population density described here, fresh mushrooms would be among the commodities which support growth of *L. monocytogenes* to a higher extent. The growth rate and maximum population densities attained in the current trials were similar to those described for brocoli and asparagus and higher than those reported for the rest of the produce analysed by Hoelzer and co-authors [19].

ComBase predictions under temperature, pH and a_w_ conditions prevailing in the current mushroom challenge tests also foresee growth of *L. monocytogenes* to similar numbers as were seen in the current study (Figure 3). However, some deviations between ComBase predictions and the experimental results could be observed. ComBase predicted slightly higher growth rates (e.g., 0.11 log CFU/g/h at 15 °C; Figure 3). ComBase also predicted *L. monocytogenes* growth under temperature, pH and a_w_ conditions prevailing in the mushroom casing challenge tests, while survival without overall increase in numbers was observed (Figure 1). As previously mentioned, this may be due to the high numbers of background microflora which can compete with *L. monocytogenes*. Predictive models used by ComBase Predictor tool are based only on output from laboratory experiments observed in culture media under well controlled laboratory conditions and do not take into account the effect background microflora may have. Divergences from ComBase predictions underline the importance of conducting challenge tests to evaluate *L. monocytogenes* growth potential in foods. 

Whereas no substantial changes in pH and moisture content were observed in mushroom casing throughout the challenge test, in whole and sliced mushrooms an increase in pH and moisture was observed. These changes in the condition of the mushrooms could be attributed to the increasing numbers of *L. monocytogenes*. However, the condition of the mushrooms was not changed enough to indicate spoilage to a consumer until very high bacterial numbers were reached. 

Although the growth rates observed were similar in whole mushrooms and sliced mushrooms, maximum population densities were higher in sliced mushrooms (9.5 log CFU/g *vs.* 7.3 log CFU/g). The differences in *L. monocytogenes* growth between sliced and whole mushrooms can be attributed to the increased available nutrients and available attachment surface of the sliced mushrooms. In addition, breakage of tissues during slicing may also make more nutrients available for use by *L. monocytogenes*.

## 5. Conclusions

In conclusion, from the sparse research previously undertaken into the relationship between *L. monocytogenes* and mushrooms [13], in addition to the findings reported here, it is clear that growth of *L. monocytogenes* is supported in mushrooms. Although mushroom casing did not support the growth of *L. monocytogenes*, it did allow the survival of cells which could then be transferred to the mushrooms during growth. 
